# Research progress on the discovery and mechanism of nutritional intervention substances for pulmonary fibrosis: a focus on astragalus and its bioactive components

**DOI:** 10.1515/biol-2025-1330

**Published:** 2026-05-25

**Authors:** Yueying Jiang, Hongfei Xing, Ling Li, Wenhua Jin, Hejing Tang, Senyang Hu, Zihao Wang, Yongting Luo, Yinhua Zhu, Qingchang Xing

**Affiliations:** Department of Traditional Chinese Medicine, The Eighth Medical Center of Chinese People’s Liberation Army (PLA) General Hospital, Beijing, 100091, China; Beijing University of Chinese Medicine, Beijing, 100029, China; Beijing Advanced Innovation Center for Food Nutrition and Human Health, Department of Nutrition and Health, China Agricultural University, Beijing, 100193, China

**Keywords:** pulmonary fibrosis, nutritional intervention, bioactive compound discovery, mechanism of action, *astragalus*

## Abstract

Pulmonary fibrosis (PF) is a chronic, progressive, and fatal fibrotic pulmonary interstitial lung disease characterized by excessive fibrosis. Its incidence is increasing globally, and the challenges associated with treatment and poor prognosis make it a significant public health burden, underscoring the urgent need for effective interventions. Nutritional interventions have garnered considerable attention for their potential to ameliorate PF. Notably, certain natural plant-derived bioactive compounds, exemplified by *Astragalus* and its constituents, demonstrate multifaceted benefits in combating PF. These include attenuating inflammatory responses, modulating immunity, exerting antioxidant effects, and promoting metabolic regulation. Through a comprehensive analysis of the existing literature, this review explores how nutritional interventions may improve the prognosis and quality of life for PF patients. It specifically focuses on discovering and summarizing the therapeutic effects of *Astragalus* and its active ingredients on PF, aiming to provide a theoretical foundation and reference for developing more effective therapeutic and management strategies for this disease.

## 1 Introduction

Pulmonary fibrosis (PF), particularly its idiopathic form (IPF), represents a chronic, progressive, and ultimately fatal interstitial lung disease characterized by the irreversible scarring of lung parenchyma [1]. This pathological remodeling leads to irreversible lung function impairment and ultimately, respiratory failure [2]. While the pathogenesis of PF is complex and multifaceted, current medicine, pirfenidone and nintedanib offer merely disease-modifying effects by slowing the rate of lung function decline, rather than halting or reversing the fibrotic process. And their utility is further constrained by significant side effects and high cost, Consequently, the discovery of novel, effective, and accessible interventions for PF is critically important.

Currently, nutritional interventions and the exploration of bioactive compounds from natural products have emerged as promising complementary approaches. Notably, *Astragalus* (Huangqi), an herb with a millennia-old history has been used in Chinese medicine to treat respiratory ailments. Modern pharmacological research has revealed that *Astragalus* is not merely a nutritional supplement but a rich source of bioactive components including Astragaloside IV (AS-IV), *astragalus* polysaccharides (APS), and flavonoids. They exhibit potent anti-inflammatory, antioxidant, immunomodulatory, and anti-fibrotic properties.

This review focuses on PF, systematically describing its core Pathogenesis. It highlights the mechanistic research on nutritional interventions and the bioactive components of *Astragalus* in treating PF, providing insights into their potential therapeutic value.

## 2 Current therapeutic options for PF are limited and non-curative

Treatment primarily relies on antifibrotic agents (pirfenidone and nintedanib) and lung transplantation [3]. While antifibrotic drugs can reduce the risk of acute exacerbations and slow the rate of lung function decline, they do not halt or reverse the disease and are associated with significant adverse effects [4]. Lung transplantation remains the only intervention capable of prolonging survival in PF, but its application is severely constrained by donor organ scarcity, high costs, and medical complexity [5]. Furthermore, PF patients frequently suffer from comorbidities such as chronic obstructive pulmonary disease (COPD), emphysema, and gastroesophageal reflux disease (GERD), which can complicate the use of antifibrotic medications [6].

## 3 Pathogenesis of PF

### 3.1 Inflammation

**Figure 1:**
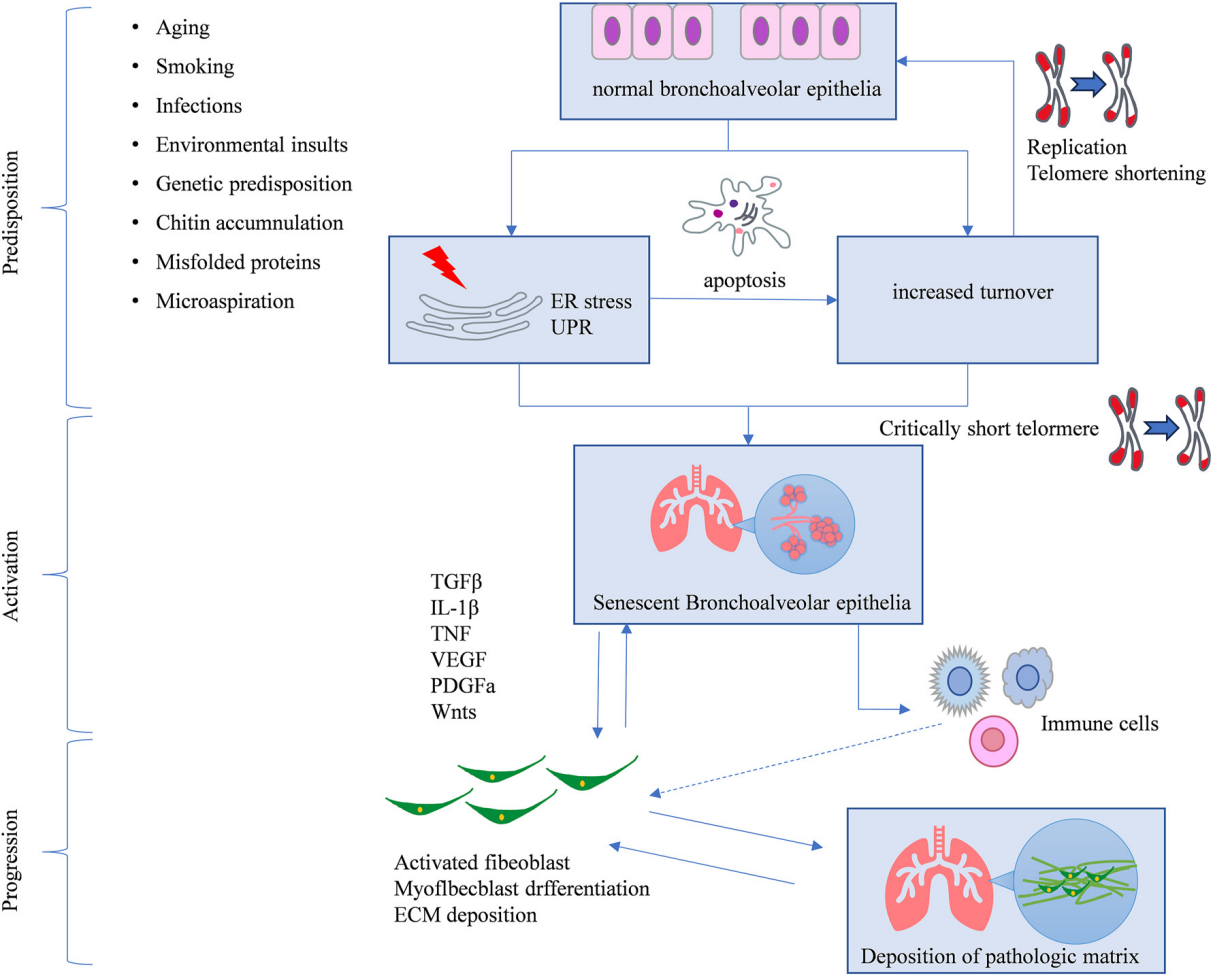
Mechanisms underlying PF development.

The pathogenesis of PF is widely believed to initiate from repetitive micro-injuries to the alveolar epithelium, coupled with aberrant repair mechanisms (Figure 1). Inflammatory cells are pivotal in disrupting the delicate balance between injury resolution and fibrosis [7]. Extensive experimental evidence confirms that the early phase of PF features prominent infiltration of inflammatory cells, particularly macrophages and lymphocytes, alongside an increase in fibroblasts. Activated macrophages secrete a plethora of pro-fibrotic cytokines, including transforming growth factor-β (TGF-β), interleukin-1β (IL-1β), and tumor necrosis factor (TNF). Macrophages also contribute to PF progression by modulating signaling pathways, which influence the TGF-β1/Smad signaling cascade, a central driver of fibrosis [8].

### 3.2 Cellular pyroptosis

Cellular pyroptosis, a lytic, pro-inflammatory form of programmed cell death, is implicated in immuno-inflammatory diseases and cancer, often exacerbating tissue damage through the induction of severe inflammation [9]. In PF, pyroptosis can be activated by inflammatory stimuli, leading to the release of pro-inflammatory cytokines (e.g., IL-1β, IL-18) and damage-associated molecular patterns (DAMPs). This amplifies the inflammatory cascade and releases mediators that drive the activation of pro-fibrotic factors. Furthermore, pyroptosis can induce EMT, thereby promoting the proliferation of fibrotic tissue within the lung [10]. Key signaling pathways mediating pyroptosis include the canonical Caspase-1-dependent pathway, the non-canonical Caspase 4/5/11 pathway, and the more recently identified Caspase-3-mediated pathway.

### 3.3 Oxidative stress

Oxidative stress, resulting from an imbalance between reactive oxygen species (ROS) production and antioxidant defenses, plays a significant role in PF pathogenesis. Excessive ROS accumulation overwhelms cellular antioxidant capacity, leading to impaired cellular functions. This creates a vicious cycle where injured lung tissue undergoes repeated, maladaptive cycles of repair and remodeling [11]. Heightened oxidative stress accelerates PF progression; individuals with PF often exhibit abnormal redox activity and are increased susceptibility to infections, further hastening disease onset. Therefore, mitigating excessive oxidative stress activation represents a potential strategy to impede PF progression, and maintaining redox homeostasis is considered a key protective factor [12].

### 3.4 Epithelial-mesenchymal transition

Epithelial-mesenchymal transition (EMT) is a critical process in fibrosis, whereby differentiated epithelial cells lose their polarity and cell-cell adhesion properties and acquire a migratory, invasive mesenchymal phenotype. During EMT in PF, alveolar epithelial cells detach, undergo cytoskeletal reorganization, and gain the ability to produce ECM components. EMT regulates and transduces signaling pathways from multiple sources including TGF-β1/Smad [13].

### 3.5 Autophagy

Autophagy, a conserved intracellular degradation pathway, plays a protective role by preventing excessive collagen accumulation; conversely, impaired autophagy exacerbates PF. Evidence indicates that autophagy is deficient and inhibited in the lung tissues of PF patients, suggesting that modulating autophagic flux represents a potential therapeutic strategy [14].

### 3.6 Cellular senescence

Cellular senescence is increasingly recognized as a major driver of IPF. Hallmarks of senescence include telomere attrition, genomic instability, mitochondrial dysfunction, epigenetic alterations, loss of proteostasis, deregulated nutrient sensing, stem cell exhaustion, and altered intercellular communication [15]. These alterations contribute to the development of a senescent phenotype in lung fibroblast, which secrete a complex mixture of factors (the senescence-associated secretory phenotype, SASP) that promote chronic inflammation, ECM remodeling, and fibrosis [16], 17]. Fibroblasts isolated from the lungs of IPF patients exhibit a heightened senescent phenotype, characterized by increased expression of classic senescence markers (p16, p21) and a pronounced pro-fibrotic secretory profile, positioning them as a key contributor to the fibrotic microenvironment [18]. The clearance of senescent cells using the senolytic cocktail dasatinib and quercetin (D + Q) reduced ECM deposition, improved lung function, and enhanced physical capacity, establishing a causal link between cellular senescence and PF pathogenesis [19].

## 4 Nutritional interventions to improve PF

### 4.1 Nutritional status of patients with PF

Nutrition is intrinsically linked to health and disease pathogenesis. Epidemiologic studies confirm that unhealthy dietary patterns increase the risk of age-related chronic diseases and accelerate mortality [20]. Conversely, healthy dietary patterns can delay or prevent such conditions, promoting longevity [21]. Emerging evidence underscores the crucial role of nutritional status in PF management. PF patients frequently exhibit malnutrition, characterized by weight loss, muscle wasting, and immune dysfunction, especially in IPF and cystic fibrosis (CF). These manifestations not only compromise overall health and quality of life but may accelerate the fibrosis progression and correlate with increased mortality [22]. Malnutrition exacerbates the PF pathology by impairing immune function, promoting inflammation, and dysregulating lung tissue repair [23]. Furthermore, PF associated inflammation elevates metabolic demands and alters nutrient utilization, establishing a vicious cycle that worsens nutritional status [24]. Adequate nutrition, however, enhances lung function, improves endurance and quality of life, and may extend survival [25], 26].

Nutritional assessment in PF should extend beyond basic anthropometrics (e.g., weight, body composition) to include detailed evaluation of protein, micronutrient, and vitamin intake. For instance, iron accumulation is implicated in PF pathogenesis, as excess iron promotes oxidative stress and inflammation, exacerbating lung tissue damage [27]. Rational nutritional interventions must therefore address these factors for comprehensive disease management.

### 4.2 Nutritional interventions and the gut-lung axis

Nutritional intervention is an established strategy for improving patient health, gaining prominence in chronic disease management. Evidence indicates that tailored nutritional support enhances physiological status, mental health and quality of life [28], serving both as a therapeutic tool and a recovery-enhancing strategy [29]. Modalities include dietary modification, nutritional supplementation, and individualized support programs, with dietitian involvement shown to significantly improve nutritional status and treatment adherence [30].

Newer studies have identified a significant interaction between the gut and the lungs, The gut-lung axis, a bidirectional communication network linking gut microbiota to lung health, has emerged as a key modulator of respiratory disease. Gut microbiota composition and function influence PF development through immune regulation, microbial metabolite signaling, and neural pathways [31]. Beyond local immune modulation, gut microbial dysbiosis has been shown to exacerbate distal fibrotic processes via systemic dissemination of microbial metabolites and endotoxins, which can prime innate immune responses and lower the threshold for fibroblast activation [32]. Gut microbial dysbiosis increases susceptibility to lung infections [33], 34]. Dietary interventions aimed at modulating the gut microbiota may therefore confer protection against PF by restoring immune homeostasis and reducing pro-fibrotic signaling [35].

### 4.3 Nutritional interventions for PF

Nutrition is integral to PF management, influencing both adjuvant therapy and disease progression. Personalized nutritional interventions improve the nutritional status, physical function and quality of life, thereby enhancing clinical prognosis. Given the roles of chronic inflammation and oxidative stress in PF, research focuses on how specific dietary patterns and key nutrients impact outcomes. Optimized nutrient intake increases muscle mass, bolsters antioxidant defenses, and reduces inflammation, contributing to improved PF management [36]. Targeted supplementation with proteins, omega-3 fatty acids, vitamins, and antioxidants, may enhance immune responses, slow disease progression, and mitigate aging-related declines [37].

Dietary patterns play an important role in the nutritional status of PF patients. Diets rich in antioxidants and anti-inflammatory components (e.g., Mediterranean diet), may preserve lung function, slow disease progression [38] and attenuate age-related immune decline [39].

Adequate protein intake ameliorates inflammation and apoptosis, promotes lung cell regeneration, reduces fibrosis extent and preserves muscle mass. Plant-based proteins are associated with a reduced inflammatory markers and may benefit PF patients [40].

Omega-3 fatty acids and their metabolites have anti-inflammatory properties, potentially attenuating inflammation-driven lung damage and fibrosis and fibrosis progression. A multi-institutional cohort clinical study found higher dietary omega-3 fatty acids intake correlated with improved oxygen-carbon dioxide exchange and longer transplant-free survival in PF patients, independent of smoking history or cardiovascular disease. Furthermore, each unit increase in plasma omega-3 index was associated with a 1.43 mL/min/mmHg improvement in DLco and a 56 % reduction in risk of death or lung transplantation. This protective effect was particularly pronounced in patients with shortened telomeres, suggesting omega-3 fatty acids may serve as a modifiable prognostic factor in PF [41].

Vitamins support immune function, antioxidant activity, and cellular processes [42]. Deficiencies in fat-soluble vitamins (A, D, E) are potential PF risk factors [42]. Vitamin D attenuates PF onset and progression by inhibiting TGF-β signaling pathway [43], with deficiency associated with exacerbation of fibrosis [23].

Compounds like vitamin C, vitamin E, and plant-derived antioxidants mitigate fibrosis by reducing oxidative stress through free radical scavenging and improved cell function [44], [45], [46]. Zinc, selenium and vitamin C enhance immune response [47]. Dietary phytoestrogens may attenuate chronic lung injury and fibrosis induced by hydrochloric acid, suggesting novel intervention avenues [48]. An antioxidant-enriched multivitamin formulation (containing β-carotene, vitamin E, coenzyme Q10, and selenium) delayed the time to first respiratory exacerbation and reduced antibiotic requirement by 50 % in patients with chronic respiratory inflammation, providing clinical evidence for antioxidant synergy in mitigating fibrotic progression [48].

## 5 Astragalus and its active ingredients in ameliorating PF

Although senolytics, including D + Q, exhibit potential for translation into clinical trials, their side effects (e.g. cytopenias) in early IPF trials are observed, emphasizing the need to develop safer alternatives like senomorphics [19]. Phytochemicals have garnered attention for their multifaceted bioactivities and potential in PF treatment. For example, natural flavonoids counteract fibrosis via antioxidant and anti-inflammatory mechanisms [49]. *Astragalus* extracts and constituents demonstrate particularly potent antifibrotic properties through diverse pathways [50]. Here we have summarized the mechanistic connections between general nutrients and *Astragalus* bioactive components (Table 1).

**Table 1:** The mechanistic connections between general nutrients and *Astragalus* bioactive components.

Pathology	Intervention strategy of general nutrients	Intervention strategy of astragalus bioactives	Connections	References
Inflammation	Omega-3 fatty acids: attenuating inflammation-driven lung damage and fibrosis and fibrosis progression.	APS: APS alleviating inflammatory responses by inhibiting the TLR4/NF-κ B signaling pathway, reducing TNF-α, IL-6, and IL-1 β levels	Omega-3 fatty acids: acting on specific downstream nuclear transcription regulatory nodes, APS: exerting a broad modulatory effect on the TLR4/NF-κB signaling pathway.	[41], 51]
Oxidative stress	Vitamin E: restoring redox homeostasis; tocotrienols: resisting oxidative stress by regulating PI3K/Akt and NF-κB pathways	Astragali Radix: regulating Nrf2/xCT/GPX4 pathway, inhibiting ferroptosis and improving PF	Vitamin E functions primarily as a direct ROS scavenger, whereas astragali Radix counteracts oxidative stress by activating the Nrf2 antioxidant pathway	[52], [53], [54]
Fibrosis-driven	Senolytics: dasatinib and quercetin selectively killing senescent fibroblasts	Astragaloside: inhibiting EMT via FOXO3a activation by blocking TGF-β1/PI3K/Akt	Senolytics: exerting anti-fibrotic effects by specifically eliminating senescent fibroblasts, APS and Vitamin D: regulating the TGF-β/Smad pathway to inhibit fibrosis	[55]
	Vitamin D: Inhibiting the TGF-*β*-SMAD signaling to suppress the EMT induced by BLM	APS: reducing TGF-β 1-induced EMT, decreasing vimentin expression and alpha smooth muscle actin.		[56], 57]
Gut microbial dysbiosis	Short chain fatty acid (Butyrate): regulating gut microbiota, inhibiting TGF-β1 response, controling macrophage differentiation	APS: improving the immunity and balance of the intestinal microbiota	APS regulate the intestinal microbiota, and metabolizing into short chain fatty acids which regulating immunity and inhibiting the TGF-*β* signaling pathway to suppress fibrosis.	[35], 58], 59]

*Astragalus*, the dried root of *Astragalus membranaceus* or *A. mongholicus* (Fabaceae), is clinically used for its “Qi-tonifying” properties in Traditional Chinese Medicine (TCM). It enters the lung and spleen meridians, exhibiting efficacy of tonifying qi and elevating yang, fixing the epidermis and stopping sweating, inducing diuresis and eliminating swelling, and generating fluids and nourishing blood [60].As a good medicine for tonifying lung qi, *Astragalus* exhibits efficacy in the treatment of cough, asthma, lung impotence and other lung diseases with high clinical safety [61]. Modern research reveals over 20 bioactive compounds, including flavonoids, saponins (e.g., AS-IV, ASI; Total Saponins of Astragalus, TSA), APS, Huangqi Glycoprotein (HQGP), and amino acids. These confer anti-inflammatory, immunomodulatory, antioxidant, and metabolic regulatory effect [62]. Bioinformatics analyses confirm *Astragalus* targets multiple PF-related pathways via multi-component synergy.

### 5.1 Main active components of *astragalus*

*Astragalus* is mainly used to improve immunity and enhance resistance to disease. Its main active ingredients include Astragalosides, polysaccharides and amino acids, which have demonstrated significant effects in anti-inflammatory, immune enhancement and tissue repair, with the advantages of safety and effectiveness (Table 2).

**Table 2:** Active components of *Astragalus* and their mechanism of action.

Active components of *astragalus*	Experimental model	Dose and administration route	Duration of administration	Mechanism of action	Evidence level	References
AS-IV	BLM-induced IPF model	20 mg/kg, by gavage	14d	AS-IV significantly inhibited TGF-β1/PI3K/Akt-induced FOXO3a hyperphosphorylation and down-regulation to reverse EMT during the progression of fibrosis	*In vivo*	[63]
APS	BLM-induced IPF model	25/50/100 mg/kg, by gavage	28d	APS attenuates bleomycin-induced PF by inhibiting TLR4/ NF-κB signaling pathway and regulating gut microbiota	*In vivo*	[59]
Flavonoids of astragalus	BLM-induced IPF model	50 mg/kg, i.p.	Every other day for 21d	Flavonoids of astragalus alleviated bleomycin-induced mouse lung fibrosis by modulating inflammation, preventing the fibrotic response and increasing epithelium regeneration	*In vivo*	[51]
AS-IV	BLM-induced IPF model	20 mg/kg, by gavage	14d	AS-IV inhibited the EMT process through the lncRNA-ATB/miR-200c/ZEB1 signaling pathway, which provides a novel approach to the treatment of IPF	*In vivo*	[64]

#### 5.1.1 Astragalus saponin

*Astragalus saponins*, extracted from the roots of *Astragalus membranaceus*, mainly including total *saponins of* TSA and AS-IV. The molecular formula of AS-IV is C_14_H_68_O_14_. Pharmacokinetic studies indicate that orally administered AS-IV is absorbed and distributes to organs including the kidneys, liver, and lungs. Moreover, the elimination half-life of AS-IV ranges from 34.0 to 131.6 min in rats and from 50.2 to 68.8 min in dogs, demonstrating its rapid clearance *in vivo* [65], 66]. Astragalus saponins have potent anti-inflammatory and antioxidant effects, inhibiting pro-inflammatory cytokine release and oxidative damage. This property makes Astragaloside show potential application in the treatment of various respiratory diseases and lays a preliminary foundation for its clinical translation.

#### 5.1.2 APS

APS is a structurally complex, water-soluble heteropolysaccharide. It is primarily composed of monosaccharides such as glucose, arabinose, rhamnose, and galactose, possessing a wide range of biological functions. The molecular weight of APS spans a broad range from 2.11 to 5,020 kDa. Notably, APS with a molecular weight around 10 kDa exhibits higher bioactivity compared to fractions exceeding 2,000 kDa. This effect can be attributed to the increased molecular size associated with higher molecular weight, which enhances transmembrane resistance, thereby impeding absorption and utilization and ultimately reducing biological activity. Then following oral administration, APS is not absorbed via the bloodstream; instead, it resists digestion in the upper gastrointestinal tract, reaches the colon, and is fermented and degraded by specific gut microbiota (e.g., *Bacteroides*, *Lactobacillus*) into metabolic products including short-chain fatty acids such as acetate, propionate, and butyrate [58], 67]. APS enhance immune response and promote lung tissue repair, which is crucial in chronic respiratory diseases. By stimulating macrophages and lymphocytes, APS enhance the body’s ability to defend itself against pathogens, boosting immune function and promoting lung repair.

#### 5.1.3 Astragalus amino acids

Various Astragalus amino acids play important roles in cellular metabolism and the synthesis of metabolism-related enzymes and hormones. For example, glutamic acid, arginine and proline support cell growth and repair. It can reduce collagen deposition by downregulating TGF-β1 and VEGF, significantly improve lung tissue structure.

### 5.2 Mechanisms of *astragalus* in ameliorating PF

#### 5.2.1 Inhibition of inflammatory response and pyroptosis

Astragaloside, polysaccharide and flavonoids inhibit inflammatory cell infiltration, suppress TGF-β1, TNF-*α* and VEGF overexpression, and reduce lung collagen in BLM-induced PF models [59]. APS alleviate inflammatory responses by inhibiting the TLR4/NF-κ B signaling pathway, reducing TNF-*α*, IL-6, and IL-1β levels and collagen deposition in lung tissue. Traditional Chinese medicine developed based on *Astragalus* significantly inhibits TLR4/NF-κ B p65/NLPR3 signaling pathway activation, reduce pro-inflammatory cytokines release, alleviate lung tissue damage, inhibit ROS and MDA formation, increase SOD activity and GSH expression [68]. TFA attenuated PF by modulating M2 macrophage polarization and suppressing TGF-β1/Smad signaling, while concurrently enhancing alveolar epithelium regeneration through upregulation of Wnt7b protein expression [68].

AS-IV protects against lung injury by suppressing the NLRP3/caspase-1-mediated pyroptosis, reducing inflammation and oxidative damage, ultimately prolonging the survival time of mice [69]. Astragaloside directly targets the key factors of cell death, decreases IL-1β and IL-18 release, reduces inflammatory cell infiltration, inhibits the transition from EMT to PF, and increases autophagic flux to improve the progression of PF [70], 71]. AS-IV (100 mg/kg) suppressed cellular senescence by downregulating p53/p21/p16 pathways, reducing SASP production and ROS generation, resulting in a 40 % decrease in lung collagen deposition and delayed EMT progression [72].

#### 5.2.2 Attenuation of oxidative stress

The levels of *α*-smooth muscle actin (α-SMA), type I collagen, fibronectin, vimentin, serum inflammatory cytokines, and oxidative stress are reduced in PF models treated with *Astragalus* and *Angelica Sinensis* [73]. *Astragalus* and *Angelica Sinensis* improve lipid peroxidation and antioxidant indicators, reduce Fe^2+^ levels, TFR1 expression, *α*-SMA and type I collagen deposition, and increase the expression of GPx4, FTH1, Nrf2 and xCT proteins. *Astragalus* and *Angelica Sinensis* ultrafiltration extract improve radiation-induced PF, by inhibiting ferroptosis via the Nrf2/xCT/GPX4 signaling pathway [54]. AS-IV (10 mg/kg, i.p.) can alleviate oxidative stress and neuronal injury. This effect is likely mediated by the activation of the Nrf2/ARE/HO-1 signaling pathway, which promotes Nrf2 production and its nuclear translocation, thereby enhancing the expression of the downstream antioxidant gene, heme oxygenase-1 (HO-1) [74].

#### 5.2.3 Suppression of epithelial-mesenchymal transition (EMT)

*Astragalus* constituents (flavonoids, polysaccharides, saponins) prevent inflammatory factors release by inhibiting TGF-β1 signaling pathway. APS improve collagen deposition and reduce fibrotic area, hydroxyproline content, and EMT markers *in vivo* and suppresses TGF-β1-induced EMT/NF-κB pathway activation *in vitro* [57]. Astragalus Saponin could control the production of the TGF-β1 downstream pathway isoform protein Smad3, which reduced the expression of related proteins in the process of EMT in PF. Astragaloside inhibit EMT via FOXO3a activation by blocking TGF-β1/PI3K/Akt, and it may attribute to the activation of long-lasting fibrotic pathway and NF-κB *in vitro*. potentially involving sirt1 AS lncRNA [75]. Treatment with APS reduce collagen deposition, fibrosis area, and hydroxyproline content in the matrix. APS significantly inhibit EMT, increase E-cadherin levels, decrease vimentin expression and alpha smooth muscle actin. In addition, APS treatment significantly reduces TGF-β one-induced EMT and NF-κB pathway activation *in vitro* [57]. In BLM-induced IPF models, AS-IV plays a protective role against PF, significantly reversing BLM induced EMT. AS-IV treatment inhibits the increase of TGF-β one and activates FOXO3a, while overexpression of FOXO3a leads to inhibition of TGF-β one-induced EMT. AS-IV therapy is similar to TGF-β one or PI3K/Akt inhibitors, which can reverse these cellular changes and inhibit EMT in A549 cells [63].

#### 5.2.4 Enhancement of autophagic and inhibition of pyroptosis

Astragaloside upregulates expression of autophagy-related proteins LC3II/LC3I and Beclin-1, inhibits Ras/Raf/MEK/ERK and PI3K/Akt/mTOR pathways, activates autophagy, degrades collagen, reduces epithelial cell senescence and myofibroblast differentiation, and improves BLM-induced ventilatory impairment in PF mice [76].

#### 5.2.5 Modulation of pulmonary neovascularization

The complexity of VEGF biology is particularly prominent, with potential non-vascular and non-angiogenic roles for VEGF in the lung, in both health and disease [77]. AS-IV normalizes hypoxia-induced HIF-1α and VEGF overexpression in human pulmonary artery smooth muscle cells [78]. Extracts from Astragaloside and *Ezhu* inhibit Lewis lung carcinoma cell growth in a xenograft mouse model by impairing mitogen-activated protein kinase signaling, VEGF production, and angiogenesis [79].

#### 5.2.6 Regulation of gut microbiota

In a BLM-induced PF mouse model, the gut microbiota composition is disrupted, exhibiting a reduction in the relative abundance of beneficial bacteria such as *Lachnoclostridium*, *Clostridium*, and *Erysipelatoclostridium*. Intervention with APS restores the abundance of these beneficial bacterial groups while simultaneously suppressing the expansion of potentially harmful bacteria [59]. Furthermore, the gut microbiota can metabolize dietary polysaccharides into beneficial metabolites such as short chain fatty acids, thereby exerting prebiotic effects. For example, butyrate, one of the metabolites of APS, has the ability of regulating gut microbiota, inhibiting TGF-β1 response, controlling macrophage differentiation and blocking the anti-fibrotic function of HDAC3 in fibroblasts [35].

## 6 Limitations and controversies

### 6.1 Model limitations

Although *Astragalus* and its bioactive components have shown great potential in preclinical studies, converting them into clinically effective intervention measures still faces significant challenges. First, the majority of the mechanism studies cited in this article rely on mouse or rat PF models induced by BLM. This model has certain differences from the chronic and progressive course of PF in human and may not fully simulate the entire picture of the disease. Moreover, most studies focus on the regulation of a single component on a specific signaling pathway (e.g., TGF-β/Smad), making it difficult to comprehensively elucidate the overall mechanism of *Astragalus* as an intervention for pulmonary fibrosis in a complex system [80].

### 6.2 Challenges of clinical transformation

To promote the clinical application of the bioactive components of *Astragalus*, the following issues need to be systematically addressed.

#### 6.2.1 Standardization of chemical composition and quality control

Future research should use standardized extracts with well-defined chemical fingerprints or highly pure monomers to ensure batch consistency and reproducibility of therapeutic effects. Studies indicate that the molecular weight of APS from different sources varies widely (2.11–5,020 kDa), and its biological activities, such as immunomodulation, are highly dependent on structural characteristics including molecular weight, monosaccharide composition, and glycosidic linkage patterns. This natural heterogeneity directly leads to fluctuations in pharmacological activity across different studies and even between batches, presenting significant challenges for the reproducibility of experimental results and the standardization of clinical products [58], 81].

#### 6.2.2 Low bioavailability

The main bioactive components of *Astragalus*, such as APS, face significant oral bioavailability challenges. The large molecular size impedes its absorption, while the strong hydrophobicity of components like flavonoids and saponins results in low bioavailability.

#### 6.2.3 Lack of clinical evidence

Currently, the supporting evidence is almost derived from cellular and animal studies. There is an urgent need for well-designed clinical trials to evaluate their safety, tolerability, and effects on lung function, quality of life, and survival in PF patients, particularly across different subtypes.

## 7 Summary and outlook

Nutritional interventions are crucial for PF management and should be integrated as complementary therapies. While current evidence supports their importance, future research must prioritize *individualized* strategies addressing patient-specific factors (e.g., PF subtype, age, comorbidities) instead of existing nutritional intervention based on general guidelines, in order to develop more targeted nutritional intervention programs [82]. Leveraging artificial intelligence and big data technologies for personalized nutritional interventions is essential for enhancing efficacy and clinical translation [83]. Therefore, exploring the application of individualized nutritional interventions in PF management, as well as more in-depth analyses of the lung and intestinal microbiomes, will refine PF therapeutic approaches.

*Astragalus* and its bioactive components demonstrate multi-target antifibrotic effects in preclinical models, including anti-inflammation, antioxidant, EMT suppression, autophagy enhancement, and angiogenesis modulation. Herein, by exploring PF pathogenesis, discovering mechanisms of *Astragalus* and its bioactive components in PF, unraveling the mechanism of action of Chinese medicine, and combining with relevant clinical cases, we can see that *Astragalus* and its bioactive components have achieved positive therapeutic effects in practical applications. In the future, it is necessary to continue in-depth research on the bioactive components of *Astragalus* and their mechanisms, providing more effective solutions for PF treatment.

Future therapeutic strategies may move beyond broad antifibrotics towards precision medicine approaches. These could include targeting specific pathological cell subpopulations identified by single-cell technologies, exploiting metabolic vulnerabilities of myofibroblasts, or using engineered agonists to promote regenerative repair without triggering fibrosis [32], as demonstrated by the targeted activation of FZD5 receptors on alveolar progenitor cells [84]. Targeting Dectin-1 with laminin or Raf1 inhibitors can alleviate fibrosis in mice and reduce pro fibrotic factors in human alveolar macrophages and fibroblasts. The multi-targeted action of natural products like *Astragalus* may synergize with the evolving paradigm [85].

PF management requires the cooperation of respiratory physicians, dietitians, psychologists and rehabilitation therapists. Multidisciplinary collaboration can provide patients with a comprehensive treatment program that ensures adequate support in nutritional, psychological, and physical aspects. Combination of nutritional interventions with pulmonary rehabilitation can significantly improve the quality of life and functional status of PF patients [86]. Therefore, establishment of a multidisciplinary cooperation mechanism to facilitate the comprehensive management of PF patients is an important way to improve patients’ quality of life and prognosis.

Clinicians should develop personalized plans through close dietitian collaboration, with regular nutritional assessment guiding intervention adjustments. Synergistic strategies combining nutrition with pharmacotherapy represent a promising frontier for improving PF outcomes.
